# Microsome Mediated in Vitro Metabolism: A Convenient Method for the Preparation of the PET Radioligand Metabolite [^18^F]FE-PE2I-OH for Translational Dopamine Transporter Imaging

**DOI:** 10.1021/acschemneuro.3c00458

**Published:** 2023-09-27

**Authors:** Magnus Schou, Nahid Amini, Akihiro Takano, Ryosuke Arakawa, Kenneth Dahl, Miklos Toth, Marie Svedberg, Andrea Varrone, Christer Halldin

**Affiliations:** † Department of Clinical Neuroscience, Center for Psychiatry Research, Karolinska Institutet and Stockholm County Council, SE-171 76 Stockholm, Sweden; ‡ PET Science Centre, Precision Medicine and Biosamples, Oncology R&D, AstraZeneca, Karolinska Institutet, S-171 76 Stockholm, Sweden

**Keywords:** PET, dopamine transporter, biotransformation, metabolism, Fe-PE2I

## Abstract

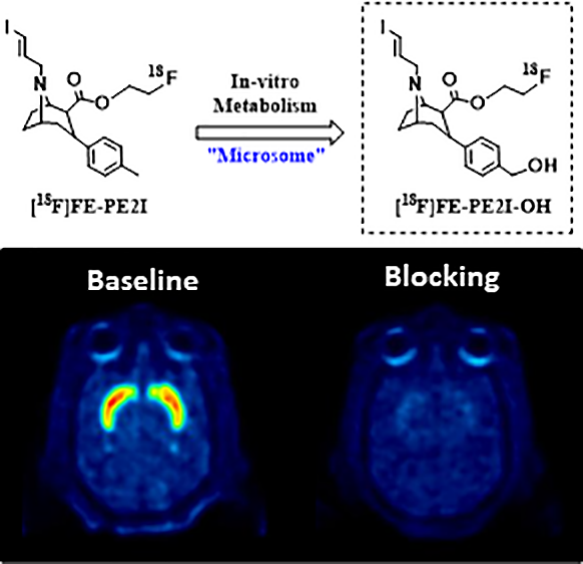

Undesired radiometabolites can be detrimental to the development of positron emission tomography (PET) radioligands. Methods for quantifying radioligand metabolites in brain tissue include ex vivo studies in small animals or labeling and imaging of the radiometabolite(s) of interest. The latter is a time- and resource-demanding process, which often includes multistep organic synthesis. We hypothesized that this process could be replaced by making use of liver microsomes, an in vitro system that mimics metabolism. In this study, rat liver microsomes were used to prepare radiometabolites of the dopamine transporter radioligand [^18^F]FE-PE2I for in vitro imaging using autoradiography and in vivo imaging using PET in rats and nonhuman primates. The primary investigated hydroxy-metabolite [^18^F]FE-PE2I-OH ([^18^F]**2**) was obtained in a 2% radiochemical yield and >99% radiochemical purity. In vitro and in vivo imaging demonstrated that [^18^F]**2** readily crossed the blood–brain barrier and bound specifically and reversibly to the dopamine transporter. In conclusions, the current study demonstrates the potential of liver microsomes in the production of radiometabolites for translational imaging studies and radioligand discovery.

## Introduction

**Figure 1:**
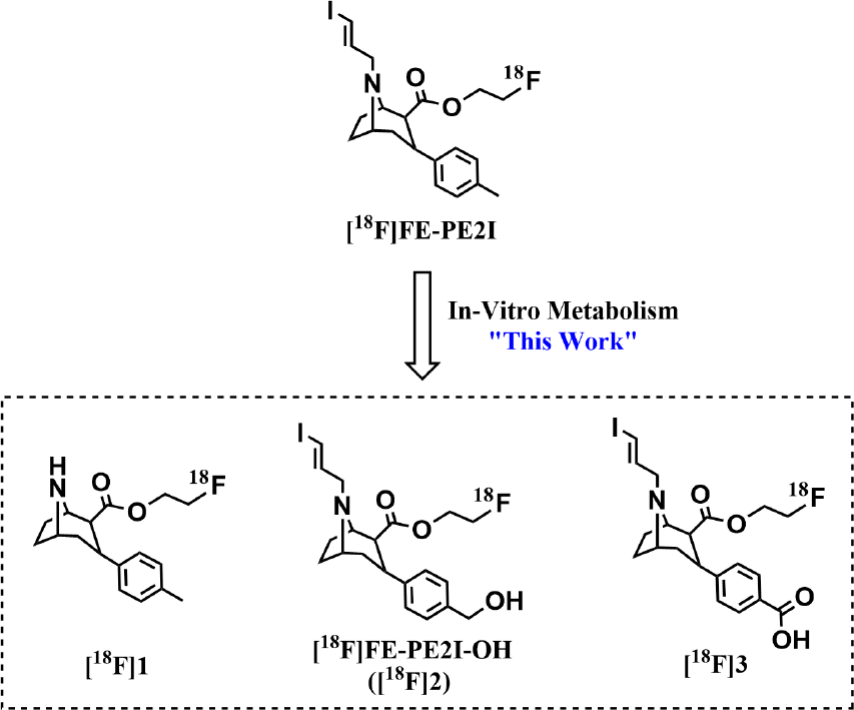
Chemical structure of [^18^F]FE-PE2I (top) and major radiometabolites observed in plasma (bottom, [^18^F]**1**–**3**).

**Figure 2:**
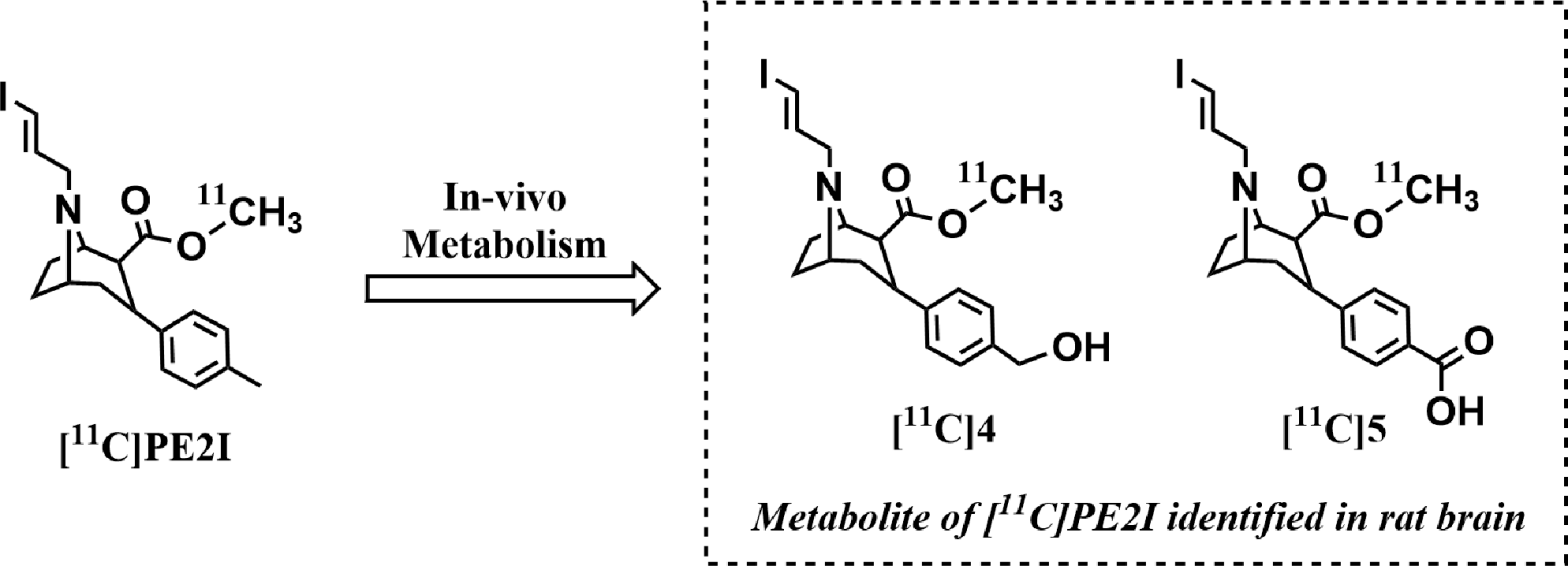
Chemical structure of [^11^C]PE2I and the two known radiometabolites observed in the rat brain ([^11^C]**4**–**5**).

[^18^F]FE-PE2I is an established radioligand for imaging the dopamine transporter (DAT) in human brain using positron emission tomography (PET).^1−5^ We have exploited liver microsomes and liquid chromatography coupled with tandem mass spectrometry in the identification of positron emission tomography (PET) radioligand metabolites of the central nervous system (CNS) in plasma.^6^ During its application to studies of [^18^F]FE-PE2I (Figure 1), we noted a similar metabolic pattern as that observed for the structural analog [^11^C]PE2I (Figure 2).^1,3−5^ Given that [^11^C]PE2I is known to produce at least two radiolabeled metabolites found in the rat brain, namely, [^11^C]hydroxylated PE2I and [^11^C]carboxyl-desmethyl-PE2I (Figure 2, [^11^C]**4**–**5**),^7^ we developed an interest in molecular imaging of the major radiometabolites of [^18^F]FE-PE2I (Figure 1). Such an undertaking is far from trivial, considering the substantial chemistry resource typically required. Each radiometabolite identified as a potential radioligand for PET imaging would require chemical synthesis to obtain the corresponding reference standard and precursor material for radiolabeling. However, since PET radioligands are produced in a low chemical amount (typically <10 μg) and at high molar activity (*A*_m_), we hypothesized that liver microsomes would have the capacity to produce the desired radiometabolites (Figure 1, [^18^F]**1**–**3**) for preclinical imaging studies directly from the isolated parent radioligand [^18^F]FE-PE2I. Although two radiometabolites of [^11^C]PE2I were identified in the rat brain, it is highly unlikely that the carboxylic acid metabolite [^11^C]**5** would penetrate the intact blood–brain barrier (BBB). A more likely scenario is that [^11^C]**5** is produced within the rodent brain via [^11^C]**4** metabolism. Because of this reason, the radiometabolite [^18^F]**2** is of particular interest for further investigation with PET. The aim of this current work was thus 2-fold: (i) to study the use of preparative microsome incubations to enable the preparation of the [^18^F]FE-PE2I radiometabolite [^18^F]**2** (Figure 1, a.k.a. [^18^F]FE-PE2I-OH); (ii) to perform in vitro and in vivo translational PET imaging studies with [^18^F]**2**.

## Results and Discussion

Because liver microsomes are commercially available from several species, we started out by testing the conversion of [^18^F]FE-PE2I in the presence of rat, monkey, and human liver microsomes. After preliminary studies, it was found that rat liver microsomes produced the most rapid turnover of [^18^F]FE-PE2I, though predominantly into the undesired *N*-desalkyl derivative [^18^F]**1**. Since the *N*-dealkylation of cocaine had been shown to be mainly mediated by CYP3A,^8^ we attempted to block this pathway by adding a high concentration of the competitive inhibitor midazolam. Inclusion of midazolam suppressed the formation of the desalkyl radiometabolite and favored the formation of [^18^F]**2**. Using this procedure, it was possible to isolate [^18^F]**2** at high radiochemical purity (>99% RCP) and molar activity (*A*_m_ > 30 GBq/μmol), albeit at low radiochemical yield (RCY = 2%, relative to [^18^F]FE-PE2I at the start-of-synthesis), primarily because of losses during sample preparation and isolation. However, despite the low RCY obtained under these unoptimized conditions, the yield was sufficient for the ensuing in vitro autoradiography and translational in vivo PET imaging with [^18^F]**2** in rats and nonhuman primates (NHPs).

**Figure 3:**
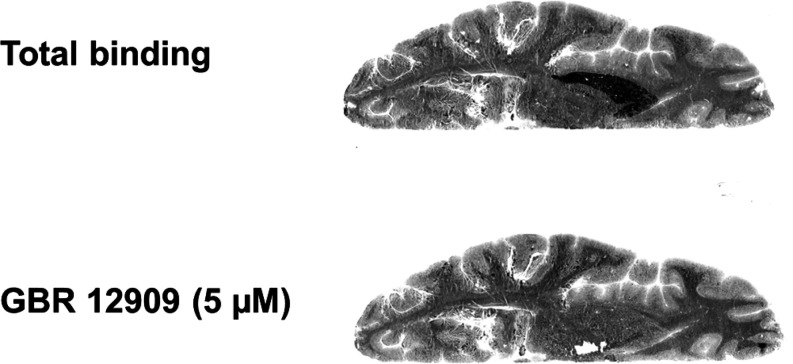
Binding of [^18^F]**2** to DAT in post-mortem human brain.

With the production procedure in hand, a series of imaging experiments with [^18^F]**2** was conducted. First, the in vitro binding to DAT was examined by autoradiography on post-mortem human brain sections. In control experiments, dense binding of [^18^F]**2** was observed in the DAT-rich striatum.^9^ This binding was completely abolished by coincubation with a high concentration (5 μM) of the selective DAT inhibitor GBR12909 (Figure 3). The nonspecific binding of [^18^F]**2** was on a similar level as that previously for [^18^F]FE-PE2I,^4^ which was not entirely unexpected given the similarity in chemical structure and physicochemical properties of the two molecules.

**Figure 4:**
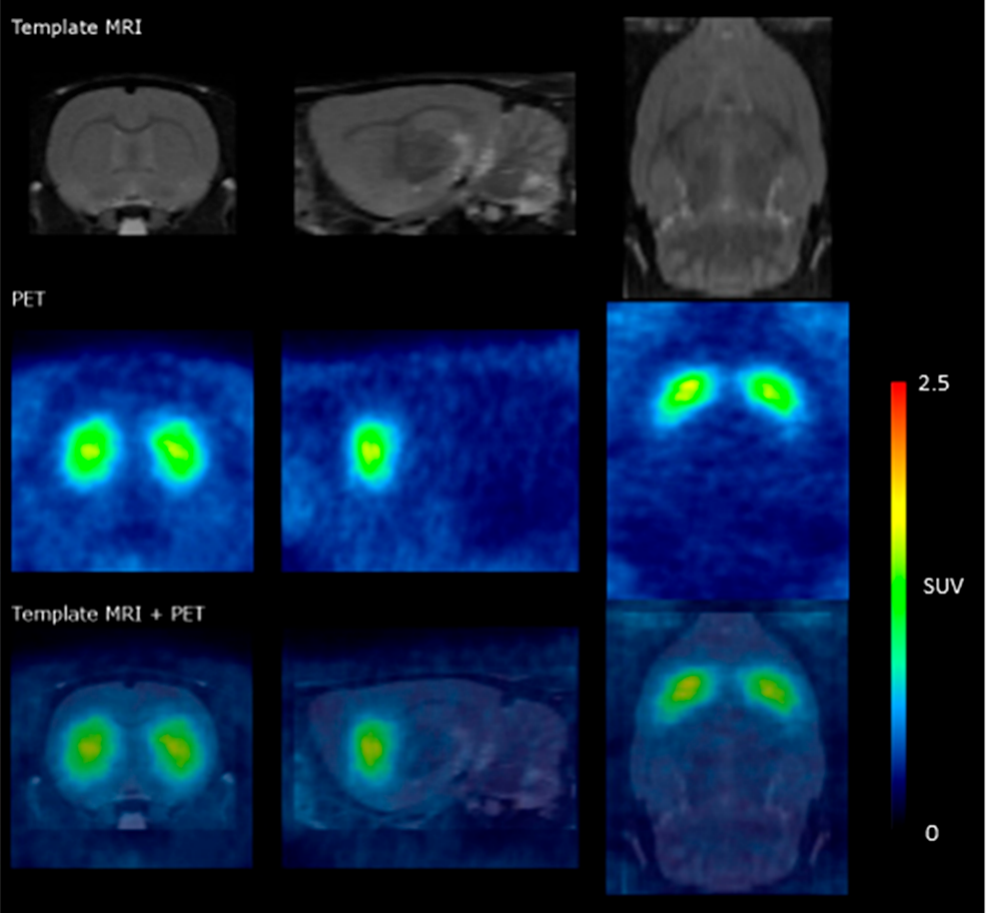
MRI and color-coded SUV PET images (average from four rats) of the rat brain obtained after the intravenous injection of [^18^F]**2**.

Though the aforementioned in vitro studies demonstrated that [^18^F]**2** binds DAT in vitro, BBB permeability of the radioligand had not been demonstrated. The first step toward establishing the brain exposure of [^18^F]**2** was in vivo PET imaging in rats. Thus, a series of dynamic baseline PET measurements were conducted following intravenous administration of [^18^F]**2**. MRI and color-coded PET images (average from four rats) are shown in Figure 4. The time-activity curve (Suppl. Figures 1–2, TACs) for brain peaked within the first 10 min at ∼4 SUV, indicating rapid brain penetration. Importantly, the TAC for the target region striatum (Caudate and Putamen) also peaked within the first 10 min (Suppl. Figure 3), after which a relatively rapid wash-out of radioactivity was observed. The cerebellum, which is a region devoid of DAT, did not retain radioactivity to a significant extent. The binding potential (BP_ND_) calculated using the simplified reference tissue method (SRTM) was 2.1 ± 0.1 (Suppl. Tab. 1).

**Figure 5:**
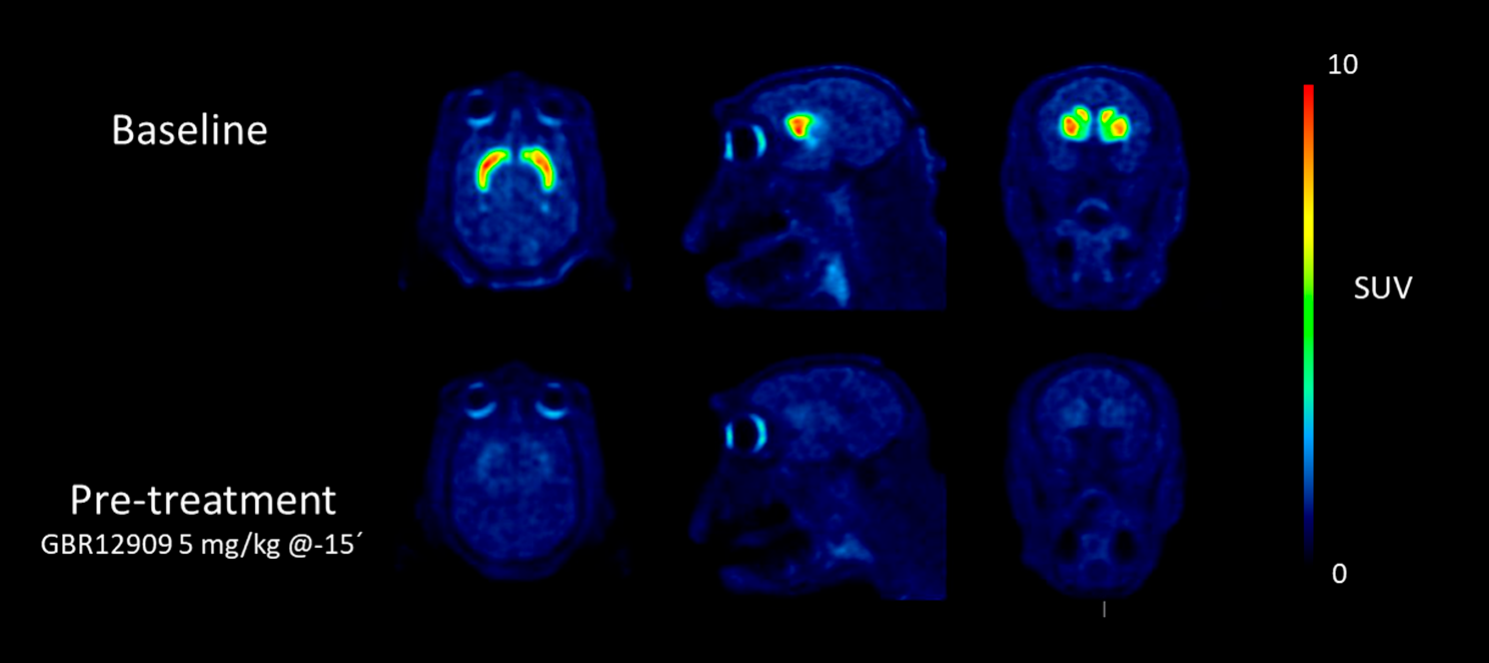
Color-coded PET images showing distribution of radioactivity in rhesus monkey brain following intravenous injections of [^18^F]**2** at baseline (top) and after pretreatment with GBR12909 (5 mg/kg) (bottom). The images represent a summary of radioactivity from 3 to 123 min after radioligand injection. Image intensity was corrected for the injected radioactivity.

A series of dynamic PET measurements was next conducted to elucidate the specificity and reversibility of [^18^F]**2** binding in the NHP brain. Following intravenous injection of [^18^F]**2**, radioactivity readily entered brain (SUV_max_ ∼ 9, Suppl. Figure 4) with the following rank order in radioactivity uptake: striatum > nucleus accumbens > thalamus ∼ substantia nigra > cerebellum (Figure 5). The observed regional distribution was consistent with the known distribution of DATs in the NHP brain and the in vitro binding of [^18^F]**2** in post-mortem human brain tissue sections.^4^ The kinetics of [^18^F]**2** uptake in the brain was relatively rapid and dependent on regional DAT density. In high density regions (e.g., striatum), the radioactivity peaked between 30 and 40 min, whereas radioactivity in the DAT-devoid cerebellum peaked within the first ten min after radioligand injection (Suppl. Figure 5). Wash-out of radioactivity from striatum was relatively rapid, with approximately 50–67% of the radioactivity remaining in tissue at the end of the PET measurement compared to peak radioactivity.

The time course for unchanged [^18^F]**2** in NHP plasma was studied using radio-HPLC. Turnover of [^18^F]**2** was rapid with between 20% and 30% parent remaining in plasma at 20 min post injection (Suppl. Figure 6). From a qualitative perspective, most of the radioactivity in plasma was polar and eluted with the void volume of the HPLC column (Suppl. Figure 7). One may speculate that this radioactivity is constituted by conjugation products of phase 2 metabolism. However, no further efforts were put into their identification since these are unlikely to pass the BBB and substantially contribute to brain radioactivity. The same applies for the acid radiometabolite [^18^F]**3**, which comprised as much as 50% of plasma radioactivity toward the end of the PET measurement. In this context, it is worth noting that negligible levels of [^18^F]**2** have been observed in NHP and human plasma following the injection of [^18^F]FE-PE2I,^1^ suggesting that [^18^F]**2** has a minor impact on the imaging of DAT using [^18^F]FE-PE2I in patients.

**Figure 6:**
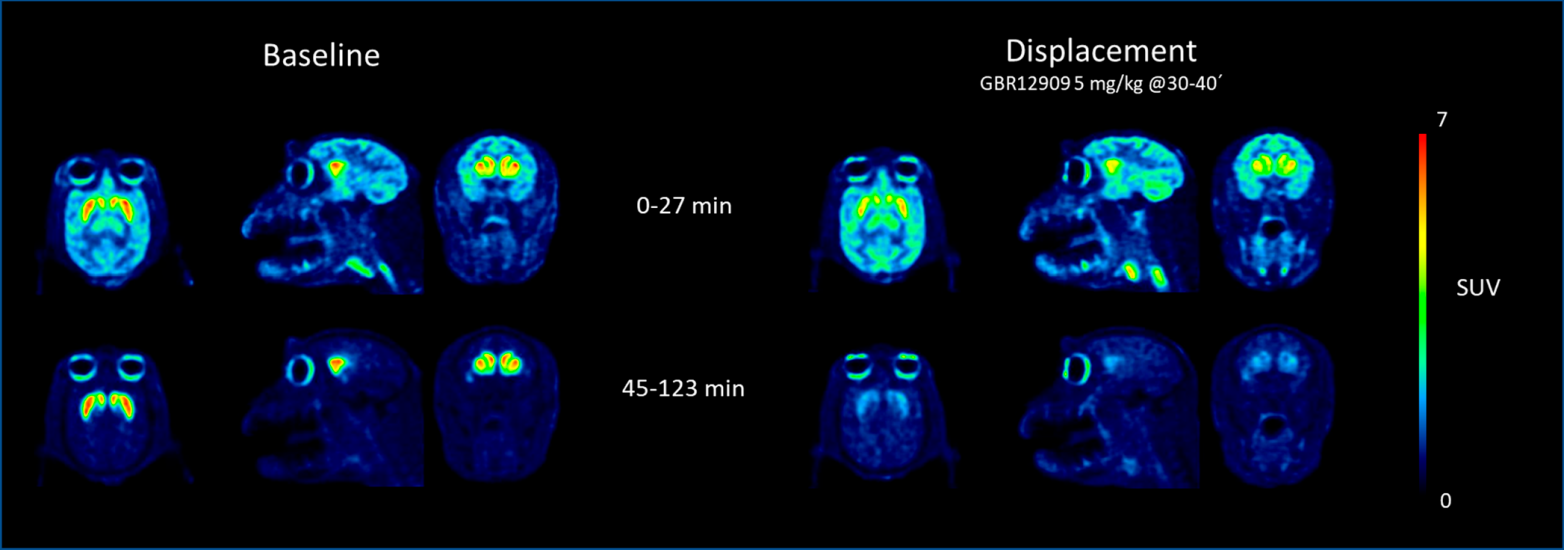
Color-coded PET images showing the distribution of radioactivity in rhesus monkey brain following intravenous injections of [^18^F]**2** at baseline (left) and after displacement with GBR12909 (5 mg/kg) (right).

In a pretreatment experiment, in which the selective DAT inhibitor GBR12909 (5 mg/kg) was infused 15 min prior to injection of [^18^F]**2**, the radioactivity was markedly reduced in all examined regions to the level of cerebellum (Figure 5, Suppl. Figure 8). Importantly, the radioactivity in cerebellum was relatively unaffected under these conditions (<10% decrease in the AUC), thus qualifying it as a suitable reference region for free and nonspecific binding in brain. A similar inhibition of DAT was observed in a displacement experiment, in which GBR12909 (5 mg/kg) was infused between 30 and 40 min after radioligand injection. In this experiment, the infusion of GBR12909 chased out [^18^F]**2** from DAT-rich regions until a homogeneous distribution of radioactivity was observed in brain (Figure 6, Suppl. Figure 9). This set of experiments thus supports the specificity and reversibility of [^18^F]**2** to DAT in the NHP brain in vivo.

The binding of [^18^F]**2** was quantified by using compartmental analysis. It was found that two compartments (2-TC) were required to adequately describe the uptake of [^18^F]**2** in brain. It is worth noting that a model in which a sum of [^18^F]**2** and the acid metabolite [^18^F]**3** was used as input function resulted in a poor fit of the TACs for striatum and cerebellum (Suppl. Figure 10). The total volume of distribution (*V*_T_) obtained from the 2-TC model was between 52 and 58 mL/cm^3^ in the striatum and 8 mL/cm^3^ in the cerebellum. The nondisplaceable binding potential (BP_ND_), calculated using cerebellum as reference, was between 8.4 and 9.4 in striatum, followed by 2.8 in nucleus accumbens. Lower binding potentials were observed in the substantia nigra (1.2) and thalamus (0.5).

Since the cerebellum had been identified as a suitable reference region, the binding of [^18^F]**2** was also quantified using SRTM. BP_ND_ values obtained by SRTM were numerically lower but correlated well with those obtained by 2-TC (*R*^2^ = 0.97) and were consistent with regional DAT density. The highest BP_ND_ was observed in striatum (5.3 and 6), followed by nucleus accumbens (2.3), substantia nigra (0.8), and thalamus (0.4). In a head-to-head comparison with [^18^F]FE-PE2I, the correlation between BP_ND_ values was excellent (*R*^2^ > 0.99, Suppl. Figure 11).

BP_ND_ values in DAT-rich regions were substantially reduced following pretreatment with GBR12909. The regional occupancy under these conditions was ≥90% in striatum, substantia nigra, and nucleus accumbens. Only an 18% occupancy was observed in thalamus, which likely reflects a high level of noise and challenges the use of [^18^F]**2** for measurement of DAT in this region. Altogether, these imaging studies have shown that [^18^F]**2** possess similar PET imaging properties as [^18^F]FE-PE2I in regard to brain uptake, regional distribution, and on-target kinetics.

## Conclusions

The current study demonstrates the utility and novel application of liver microsomes for the radiosynthesis of PET radioligand metabolites for translational imaging studies. Rat liver microsomes were used to prepare radiometabolites of the DAT radioligand [^18^F]FE-PE2I. The primary investigated hydroxy-metabolite [^18^F]FE-PE2I-OH ([^18^F]**2**) was obtained in a 2% radiochemical yield and >99% radiochemical purity. In vitro and in vivo imaging demonstrated that [^18^F]**2** readily permeated the BBB and bound specifically and reversibly to the dopamine transporter. Collectively, this work demonstrates that [^18^F]**2** has the potential for DAT imaging in human subjects.

## Methods

### Radiochemistry

HPLC solvents were obtained from Fisher (Sweden). Unless otherwise stated, all other reagents and solvents were obtained from Sigma-Aldrich and used without further purification.

### General Procedure for Radiolabeling

The preparation of [^18^F]FE-PE2I and its incubation with liver microsomes (rat, monkey, and human) followed previously published procedures.^6,10^ After the reaction was completed, proteins were precipitated by the addition of an equal volume of ice-cold acetonitrile, after which the slurry was filtered through a frit inserted in an empty solid phase extraction column. Purification was performed on a semipreparative HPLC column (XBridge, 5 μm, 250 × 50 mm, Waters) eluted with a stepwise gradient between acetonitrile and aqueous formic acid (0.1%): 10–60% over 15 min. Radiotracers were isolated from the HPLC eluent using solid phase extraction (1 cm^3^ of Oasis HLB, Waters) and eluted with EtOH (0.5 mL) into sterile saline (5 mL). The final product was sterilized via membrane filtration (Millipore, 0.22 μm). Between 0.8 and 2.4 GBq of [^18^F]FE-PE2I was used in the incubations, resulting in between 10 and 82 MBq of [^18^F]FE-PE2I-OH.

### In Vitro Autoradiography

Studies including human brain tissue were approved by the Ethics Committee at Karolinska Institutet (registration no. 03-767). The human brain used in this study was obtained from the National Institute of Forensic Medicine, Karolinska Institutet (Stockholm, Sweden). The brain had been removed at clinical autopsy and was handled in a manner similar to that previously described.^11^ Horizontal sections, including the striatum, were selected for the binding experiments.

The sections were incubated for 20 min at RT with 4 MBq of each radioligand in a TRIS buffer (50 mM; pH 7.4) containing sodium chloride (300 mM), potassium chloride (5 mM), and ascorbic acid (0.1% (w/v). The sections were then washed (same buffer) three times for 5 min each and briefly dipped in cold distilled water before being exposed to the Kodak Biomax MR film overnight. Nonspecific binding was estimated by simultaneous incubation with GBR 12909 (10 μM).

### PET Imaging in Rats

All animal experiments were conducted according to the appropriate Swedish regulations with the approval of the Animal Research Ethics Committee of the Swedish Animal Welfare Agency (Northern Stockholm Region) and were performed according to the guidelines of Karolinska Institutet regarding working with experimental animals (Dnr N210/10).

Four male Sprague–Dawley rats (490–570 g) were injected intravenously with [^18^F]**2** (9–13 MBq). Dynamic in vivo PET was acquired on a nanoPET (Mediso) system over 93 min. A preliminary quantification of the binding in striatum was performed using the simplified reference tissue model (SRTM).

### PET Imaging in Nonhuman Primates

The study was approved by the Animal Ethics Committee of the Swedish Animal Welfare Agency (Dnr 145/08, 399/08, and 386/09) and was performed according to the “Guidelines for planning, conducting and documenting experimental research” (Dnr 4820/06-600) at the Karolinska Institutet, the “Guide for the Care and Use of Laboratory Animals”,^12^ the AstraZeneca bioethics policy, and the EU Directive 2010/63/EU.

Radioactivity in the brain was measured with the Siemens Molecular Imaging high resolution research tomograph (HRRT) system. The HRRT system consists of 8 panel detectors with an octagonal configuration. A transmission scan was acquired for 6 min using a single ^137^Cs-source immediately before injection of [^18^F]**2**. Images were reconstructed with Ordinary Poisson-3D-Ordered Subset Expectation.^13^

Five PET measurements were performed in two female rhesus monkeys. Session 1: PET measurements were made with 82 MBq of [^18^F]**2** in rhesus monkey 1 (5.9 kg). Session 2: PET measurements were with 45 MBq of [^18^F]**2** in rhesus monkey 2 (6.1 kg). Session 3: PET measurements were with 65 MBq of [^18^F]**2** in rhesus monkey 1. The emission measurement started 15 min after a 10 min intravenous infusion of GBR12909 (5 mg/kg). Session 4: PET measurements were with 80 MBq of [^18^F]**2** in rhesus monkey 1 (6.1 kg), followed by a 10 min intravenous infusion of GBR12909 starting at 30 min post-tracer injection. Session 5: PET measurements were with 133 MBq of [^18^F]FE-PE2I in rhesus monkey 1.

A head fixation system was used to secure a fixed position of the monkey’s head throughout the PET measurements undertaken in each experimental session.^14^ In each PET experiment, the radioligand was formulated in sterile physiological phosphate buffer (pH 7.4) solution containing 5% ethanol and injected as a bolus into a sural vein during 5 s with the simultaneous start of PET data acquisition. Radioactivity in the brain was measured continuously for 123 min according to a preprogrammed series of 34 frames.

### Analysis of Radioactive Metabolites in NHP Plasma

After intravenous radioligand administration, arterial and/or venous blood samples were collected in heparin-treated syringes at the prespecified time points. The blood samples were centrifuged at 2500*g* for 2 min to separate plasma. The supernatant plasma samples (0.4–1.6 mL) were mixed with acetonitrile (1.4× plasma volume). The resulting denatured protein emulsion was stirred with a vortex mixer and centrifuged at 2000*g* for 4 min. After addition of water (2 to 3 mL) to the supernatant plasma–acetonitrile mixture, the mixture was subsequently injected into the radio-LC system and analyzed using the chromatographic method reported previously for [^18^F]FE-PE2I.^6^

## Supporting Information Available

Analytical and preparative HPLC chromatograms; results from microsome incubations; whole brain and regional time-activity curves (TACs); PET quantification data and metabolite analysis (PDF)
