# Supplementary material for: Microsome Mediated in Vitro Metabolism: A Convenient Method for the Preparation of the PET Radioligand Metabolite [18F]FE-PE2I-OH for Translational Dopamine Transporter Imaging
Source: ACS Chem Neurosci. 2023 Sep 27;14(20):3732–6. doi: 10.1021/acschemneuro.3c00458 (PMC10587862; doi:10.1021/acschemneuro.3c00458)
Supplement: Supplementary file 1 — cn3c00458_si_001.pdf [file cn3c00458_si_001.pdf]

Supplemental Information

Microsome Mediated In Vitro Metabolism: A convenient method for the preparation of the PET radioligand metabolite $[^{18}F]$FE-PE2I-OH for translational dopamine transporter imaging

Magnus Schou[†‡*], Nahid Amini[†], Akihiro Takano[†], Ryosuke Arakawa[†], Kenneth Dahl[†‡], Miklos Toth[†], Marie Svedberg[†], Andrea Varrone[†], Christer Halldin[†]

[†]Department of Clinical Neuroscience, Center for Psychiatry Research, Karolinska Institutet and Stockholm County Council, SE-171 76 Stockholm, Sweden

[‡]PET Science Centre, Precision Medicine and Biosamples, Oncology R&D, AstraZeneca, Karolinska Institutet, S-171 76 Stockholm, Sweden.

*Corresponding author: magnus.schou@astrazeneca.com

Contents

SUPPLEMENTAL FIGURE 1. Semi-preparative radiochromatograms with and without midazolam in the microsome incubate. 3

Supplemental Table 1. Unchanged radioligand (%) over time in microsome incubations in rat, monkey and human liver microsomes. 4

SUPPLEMENTAL FIGURE 2. Individual TACs following intravenous injection of [^{18}F]**2** in rats. 5

SUPPLEMENTAL FIGURE 3. Grouped TACs following intravenous injection of [^{18}F]**2** in rats. 6

SUPPLEMENTAL FIGURE 4. Time-course for specific binding (CaudatePutamen-Cerebellum) following intravenous injection of [^{18}F]**2** in rats. 7

SUPPLEMENTAL Table 2. BP_{ND} in rat striatum following injection of [^{18}F]**2**. 8

SUPPLEMENTAL FIGURE 5. TACs following intravenous injection of [^{18}F]FE-PE2I-OH ([^{18}F]**2)** and [^{18}F]FE-PE2I in rhesus monkey. 9

SUPPLEMENTAL FIGURE 6. Time-course for specific binding ratio (A) and specific binding (B) following intravenous injection of [^{18}F]**2** in rhesus monkey at baseline (blue) and displacement conditions (red). The arrow denotes start of the GBR12909 infusion (10 min, 5 mg/kg). 10

SUPPLEMENTAL FIGURE 7. Time-course for unchanged [^{18}F]**2** (blue) and [^{18}F]**3** (red) in plasma following intravenous injection of [^{18}F]**2** in rhesus monkey. 11

SUPPLEMENTAL FIGURE 8. Radio-chromatograms of extracted plasma at two time-points following intravenous injection of [^{18}F]**2** in rhesus monkey at baseline and blocking conditions. Arrows indicate the identity of the labeled metabolites. 12

SUPPLEMENTAL FIGURE 9. TACs following intravenous injection of [^{18}F]**2** in rhesus monkey at baseline and blocking conditions. 13

SUPPLEMENTAL FIGURE 10. TACs from baseline and displacement experiments with [^{18}F]**2** in rhesus monkey. The arrow denotes the start of drug infusion (GBR12909 5 mg/kg), which took place between 30-40 min. 14

SUPPLEMENTAL FIGURE 11. Fitting TACs to metabolite corrected arterial input function 15

SUPPLEMENTAL FIGURE 12. Correlation between BP_{ND}-estimates obtained with 16

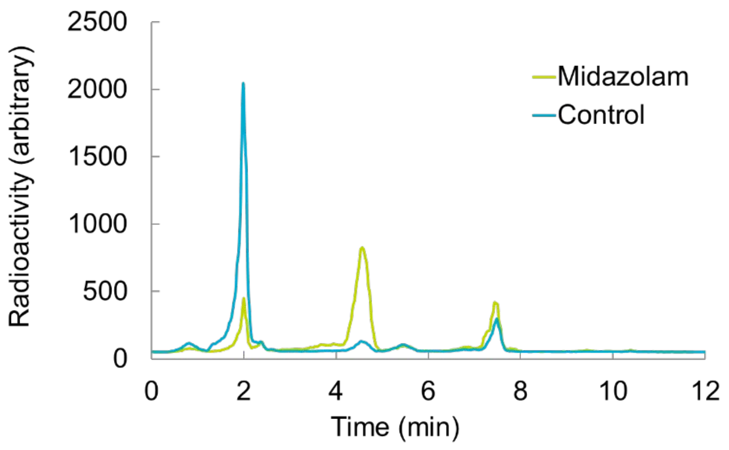

SUPPLEMENTAL FIGURE 1. Semi-preparative radiochromatograms with and without midazolam in the microsome incubate.

Data File C:\CHEM32\1\DATA\18FEPE2I\1211008000019.D
Sample Name: 18FEPE2I-OH formulation

===

Acq. Operator : RN
Acq. Instrument : Instrument 1 Location : -
Injection Date : 11/8/2012 3:32:21 PM
Inj Volume : No inj
Acq. Method : C:\CHEM32\1\METHODS\18F-FE-PE2I FAST.M
Last changed : 11/8/2012 3:31:42 PM by RN
Analysis Method : C:\CHEM32\1\METHODS\18F-FE-PE2I.M
Last changed : 2/17/2012 6:28:43 PM by NaNa
Additional Info : Peak(s) manually integrated

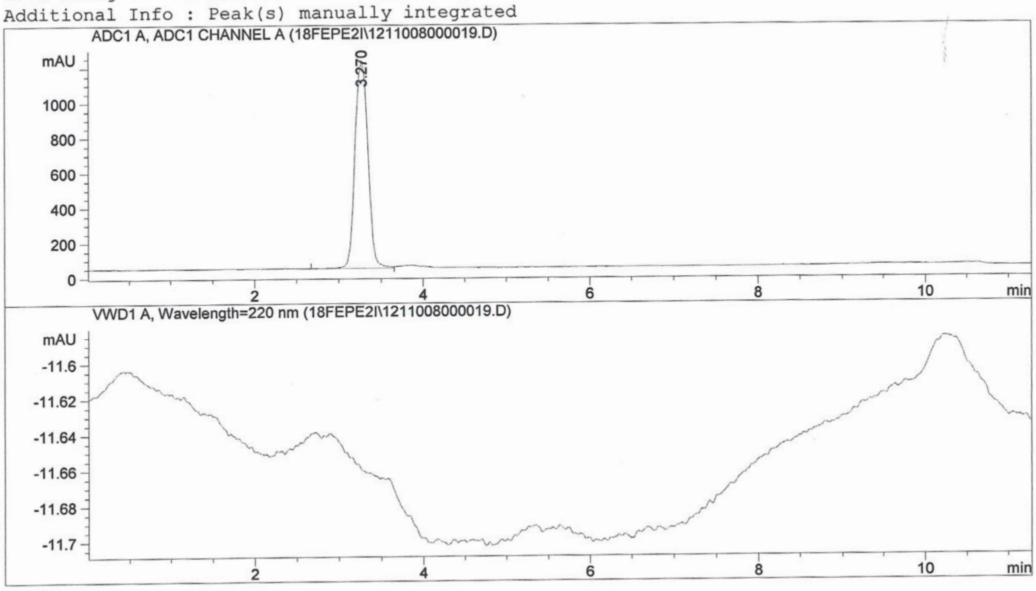

===

Fraction Information

===

Fraction collection off

===

No Fractions found.

===

===

Area Percent Report

===

Sorted By : Signal
Multiplier: : 1.0000
Dilution: : 1.0000
Use Multiplier & Dilution Factor with ISTDs

Signal 1: ADC1 A, ADC1 CHANNEL A

Peak #	RetTime [min]	Type	Width [min]	Area [mAU*s]	Height [mAU]	Area %
1	3.270	BV	0.1735	1.25767e4	1188.26343	100.0000

Totals : 1.25767e4 1188.26343

rument 1 8/21/2023 3:40:55 PM YK/JIA Page 1 of 2

SUPPLEMENTAL FIGURE 2. Radio-HPLC chromatogram of formulated solution of [^{18}F]**2** for injection.

Supplemental Table 1. Unchanged radioligand (%) following incubation of [^{18}F]FE-PE2I in rat (RLM), monkey (MLM) and human liver microsomes (HLM).

Time (min)	RLM	MLM	HLM
1	17	23	25
5	0.6	11	8
10	ND	ND	1.4
20	ND	ND	ND

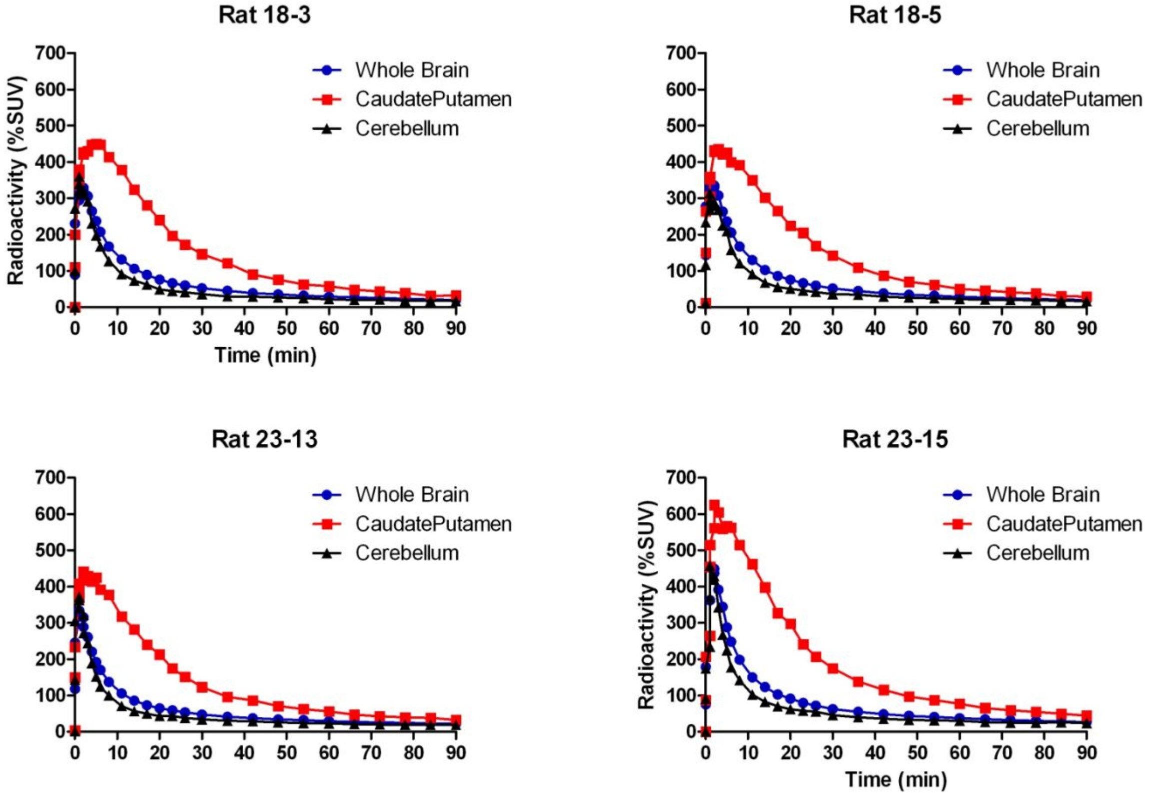

SUPPLEMENTAL FIGURE 3. Individual TACs following intravenous injection of [^{18}F]**2** in rats.

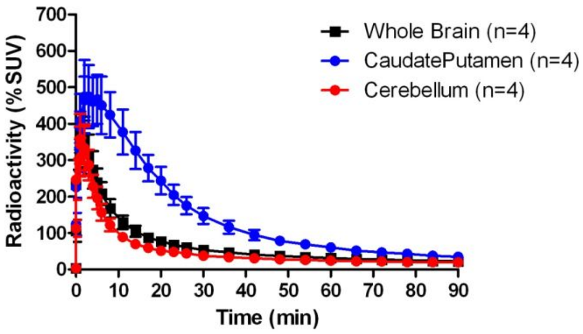

SUPPLEMENTAL FIGURE 4. Grouped TACs following intravenous injection of [^{18}F]**2** in rats.

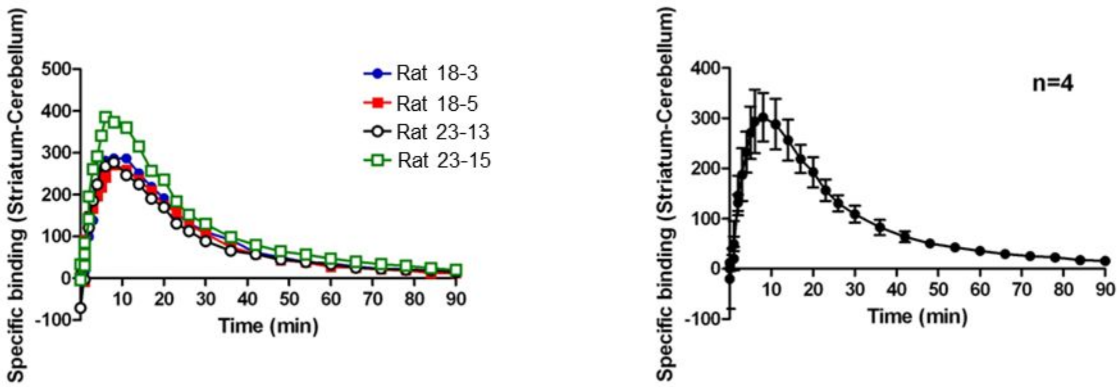

SUPPLEMENTAL FIGURE 5. Time-course for specific binding (CaudatePutamen-Cerebellum) following intravenous injection of [^{18}F]**2** in rats.

Animal ID	BP_{ND}
Rat 18-3	2.07
Rat 18-5	2.02
Rat 23-13	2.16
Rat 23-15	2.23
**Average ±SD**	**2.12±0.09**

SUPPLEMENTAL Table 2. BP_{ND} in rat striatum following injection of [^{18}F]**2**.

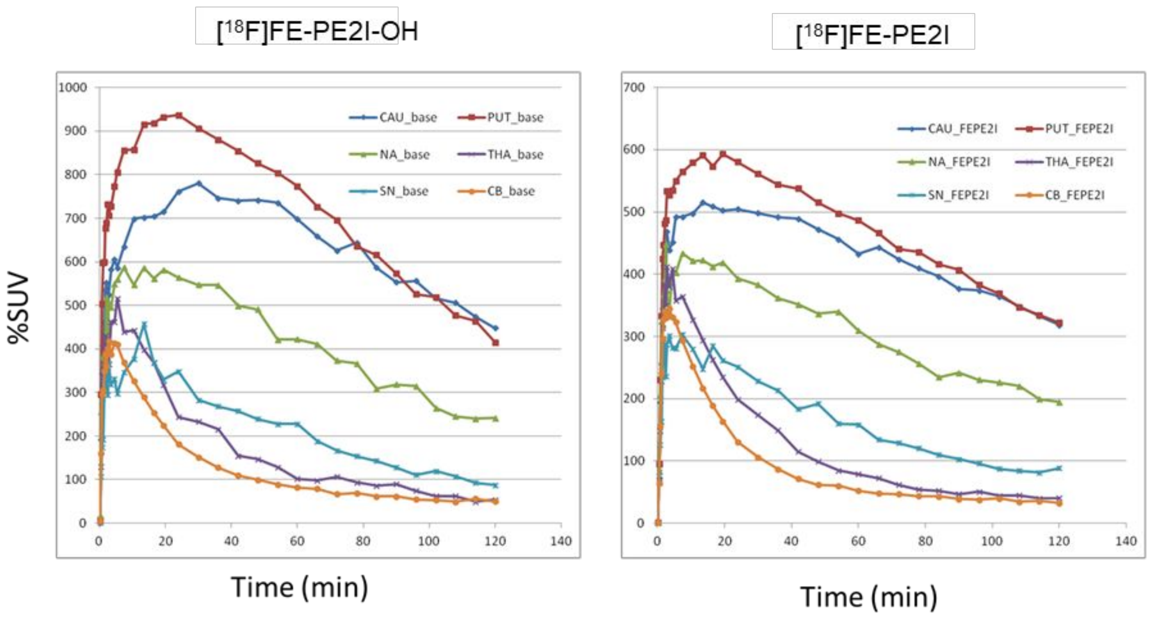

SUPPLEMENTAL FIGURE 6. TACs following intravenous injection of [^{18}F]FE-PE2I-OH ([^{18}F]**2)** and [^{18}F]FE-PE2I in rhesus monkey.

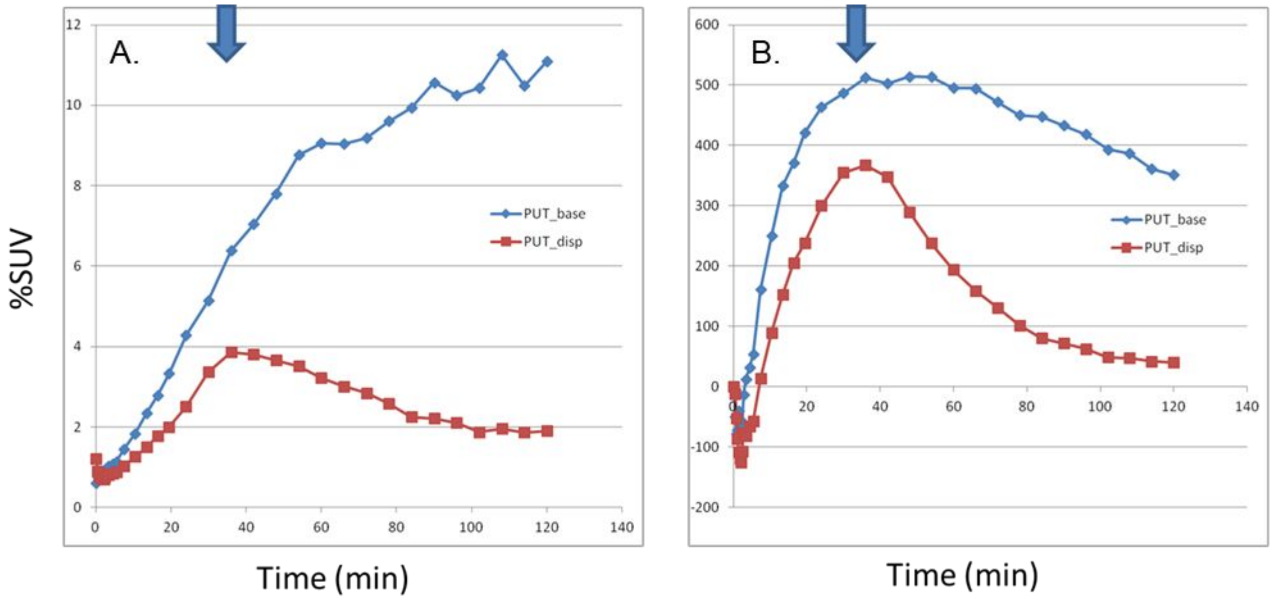

SUPPLEMENTAL FIGURE 7. Time-course for specific binding ratio (A) and specific binding (B) following intravenous injection of [^{18}F]**2** in rhesus monkey at baseline (blue) and displacement conditions (red). The arrow denotes start of the GBR12909 infusion (10 min, 5 mg/kg).

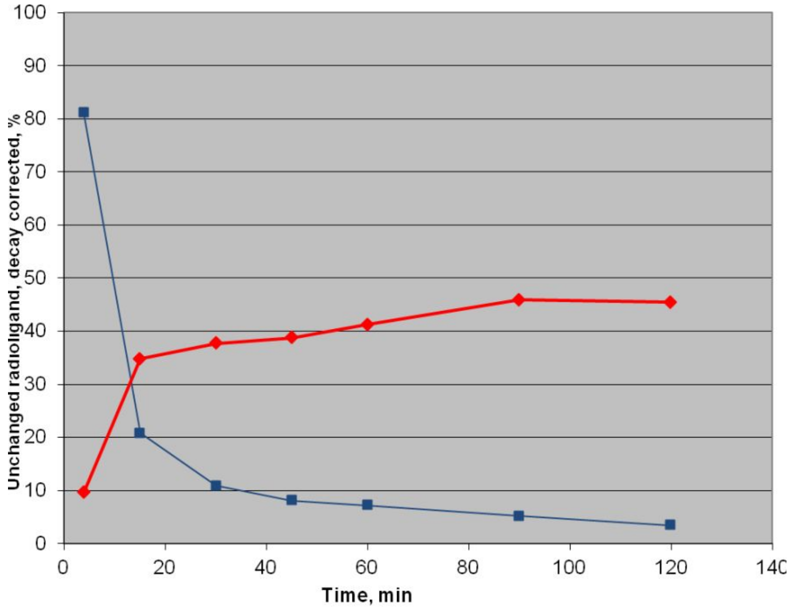

SUPPLEMENTAL FIGURE 8. Time-course for unchanged [^{18}F]**2** (blue) and [^{18}F]**3** (red) in plasma following intravenous injection of [^{18}F]**2** in rhesus monkey.

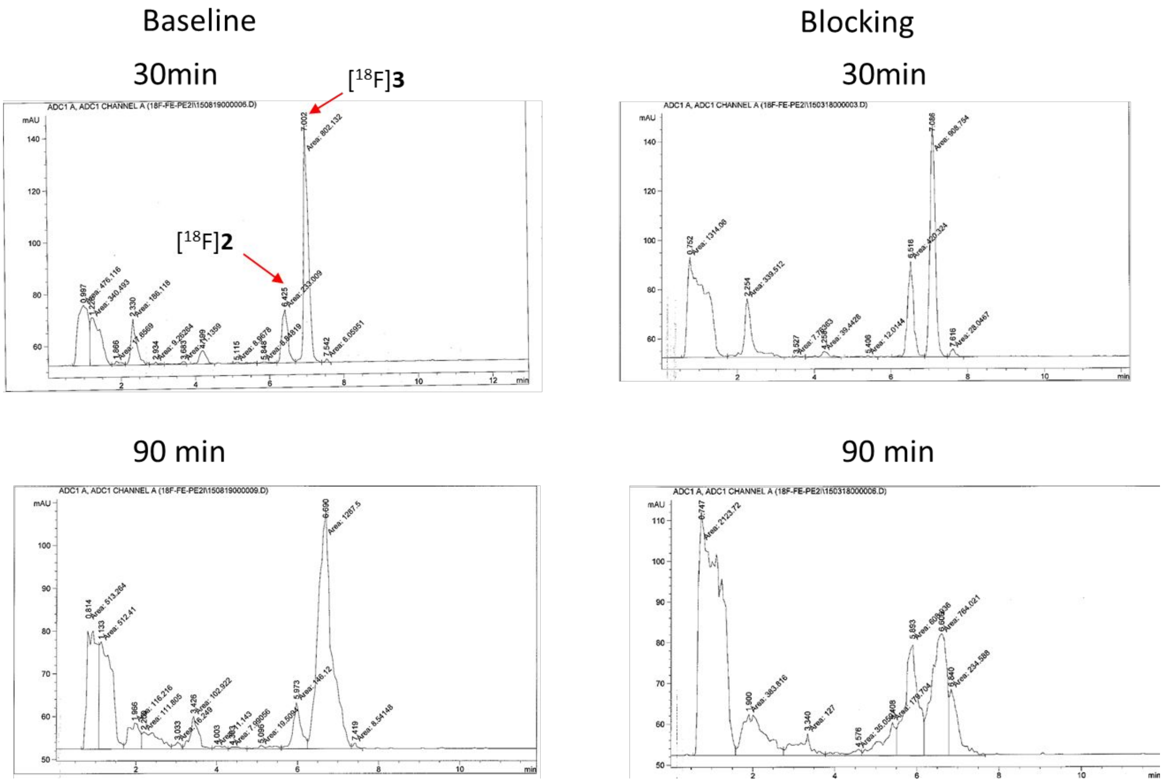

SUPPLEMENTAL FIGURE 9. Radio-chromatograms of extracted plasma at two time-points following intravenous injection of [^{18}F]**2** in rhesus monkey at baseline and blocking conditions. Arrows indicate the identity of the labeled metabolites.

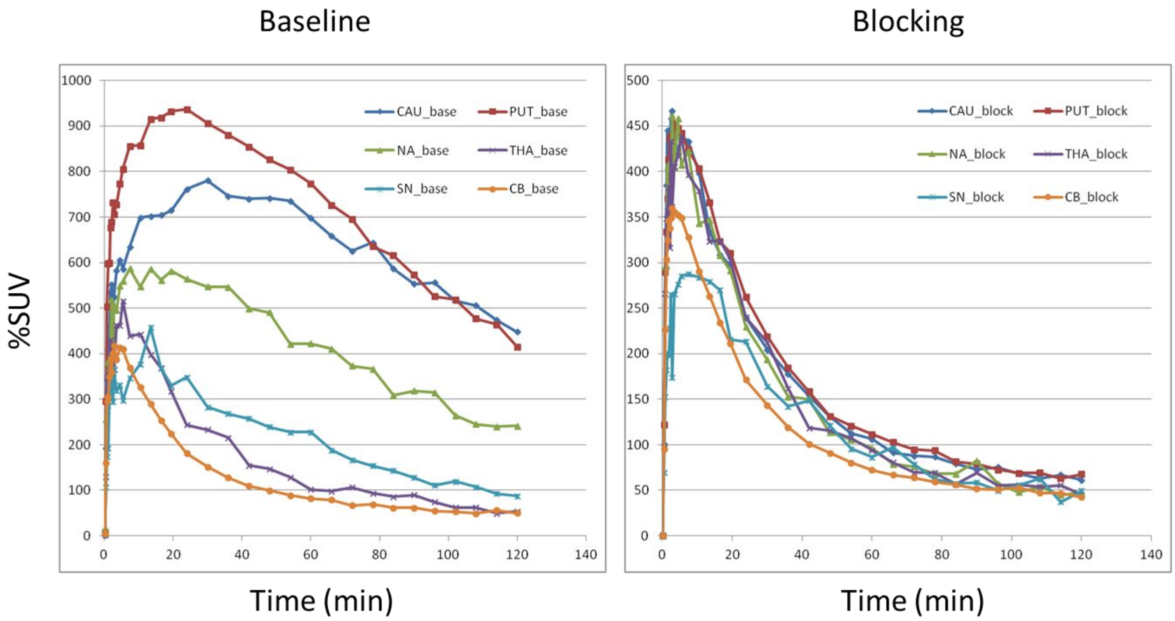

SUPPLEMENTAL FIGURE 10. TACs following intravenous injection of [^{18}F]**2** in rhesus monkey at baseline and blocking conditions.

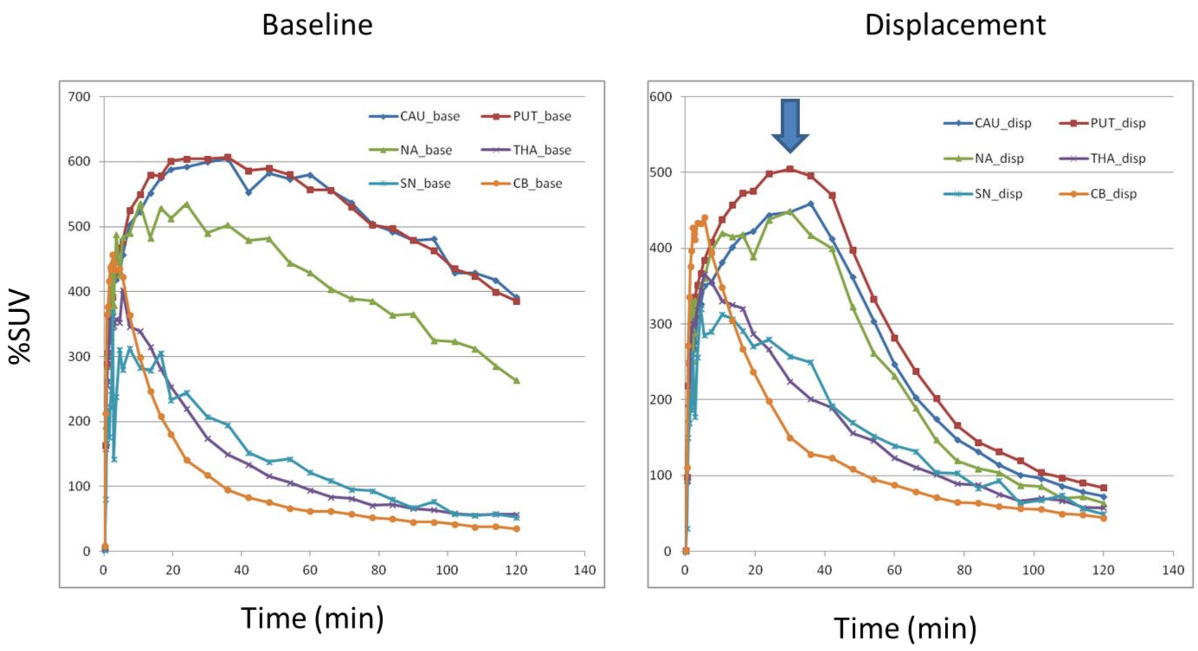

SUPPLEMENTAL FIGURE 11. TACs from baseline and displacement experiments with [^{18}F]**2** in rhesus monkey. The arrow denotes the start of drug infusion (GBR12909 5 mg/kg), which took place between 30-40 min.

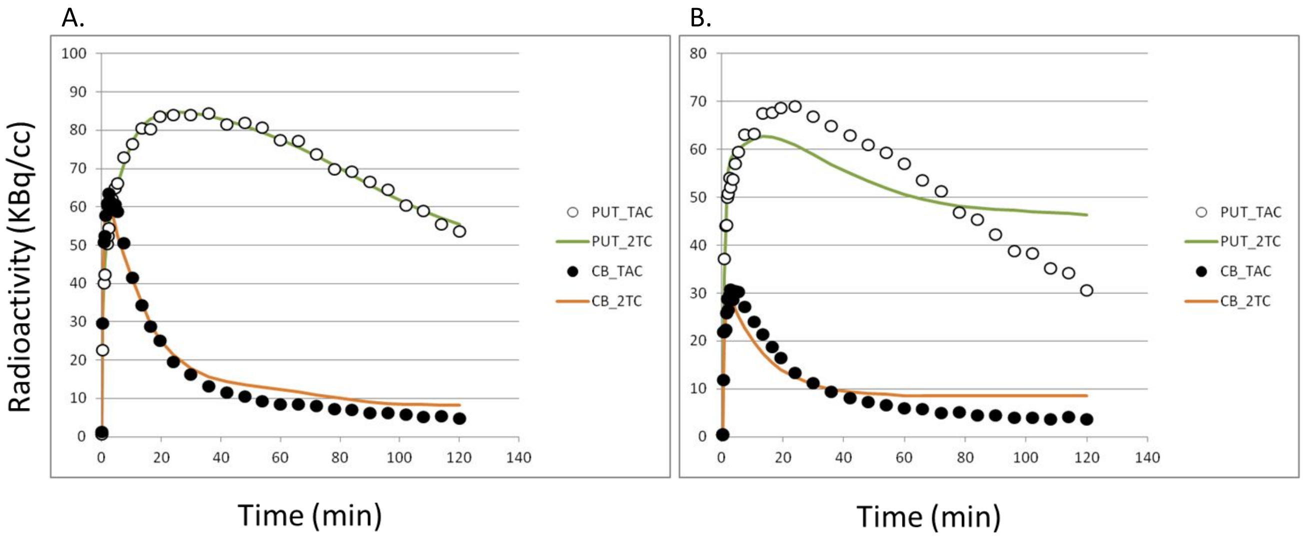

SUPPLEMENTAL FIGURE 12. Fitting TACs to metabolite corrected arterial input function in rhesus monkey for [^{18}F]**2** alone (A) or for both [^{18}F]**2** and [^{18}F]**3** (B).

BP_{ND} by SRTM

Region	[^{18}F]FE-PE2I-OH	[^{18}F]FE-PE2I
**CAU**	5.72	5.67
**PUT**	5.84	5.81
**NA**	2.79	3.21
**THA**	0.37	0.39
**SN**	0.83	1.02

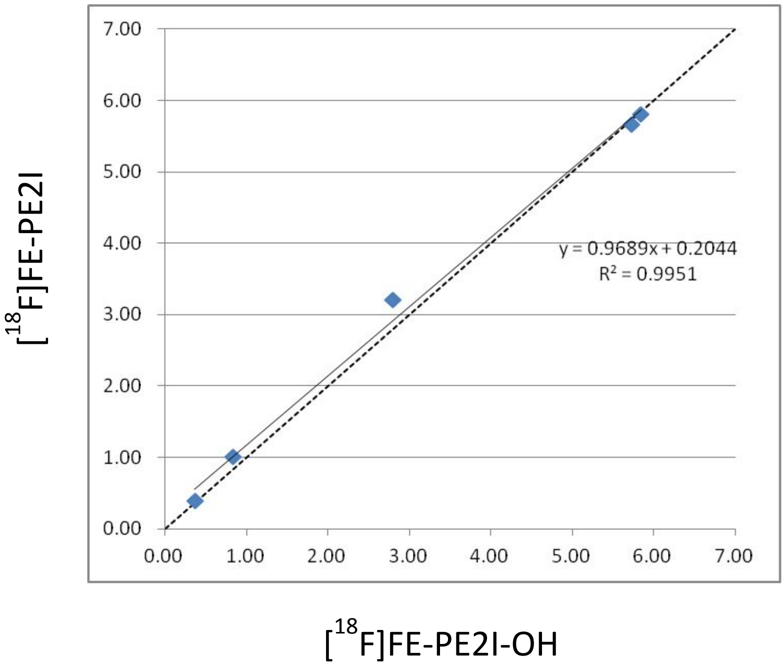

SUPPLEMENTAL FIGURE 13. Correlation between BP_{ND}-estimates obtained with [^{18}F]FE-PE2I-OH and [^{18}F]FE-PE2I in rhesus monkey.
